# Female Beauty

**Published:** 1888-03

**Authors:** 


### FEMALE BEAUTY.

The handsomest woman I ever saw was one who took great care of her health. When I knew her she was over thirty, but no girl of sixteen that I have ever seen had rosier cheeks or brighter eyes. Of course she was naturally fine looking, but the attention she gave to matters of hygiene added to and preserved her beauty. What did she do? I dont know that I can recount all, but I remember her telling me she took a sponge bath every morning; was particular about the ventilation of her apartments; took long walks when she could; ate but little meat, much fruit and cereals whenever she could get them. Another thing she did which she tried without success to get me to do; she drank her coffee without milk or cream, diluted with water.

The reason she took her coffee so was because her physicians told her it was healthier to drink it in this way. Whether the practice added to her personal charms or not I don't know. On the whole she was certainly repaid for her systematic habits, and as certainly there was nothing arduous about the performance of them. Nor was there anything bizarre about them as, it seems to me there is about the following account I read of a Chicago belle: "To keep the suppleness of her figure she stands one hour daily, fifteen minutes at a time, with her hands on her hips before a long mirror, and bending her knees out from each other she sinks slowly down to the floor as low as possible, then as slowly uprising, meantime moving her arms in any direction to their utmost length, out or up, forward or back, until when she stands erect they are ready to be placed on her hips again.

"Each movement is repeated, every time a little accelerated, until at the end of thirteen minutes it is done quickly, and a fine color is in her cheek. She then lies down on a perfectly flat couch, without a pillow, until her breath comes smooth and regular, as it will in the two minutes left in her quarter of an hour. When she plays a good deal of tennis she cuts down her exercising one-half" Of course, the benefit to be derived from this procedure is not to be questioned, whatever may be thought of it besides. It is easy to see her whole body thus receives good exercise adding to the grace of her own form, beautifying her complexion, and making her stronger and healthier,—*San Francisco Post.*

